# Assessing and reporting patient participation by means of patient preferences and experiences

**DOI:** 10.1186/s12913-020-05574-y

**Published:** 2020-07-29

**Authors:** Ann Catrine Eldh, Marie Holmefur, Kristina Luhr, Marika Wenemark

**Affiliations:** 1 Department of Health, Medicine and Caring Sciences, Linköping University, SE-581 83 Linköping, Sweden; 2 Department of Public Health and Caring Sciences, Uppsala University, Box 564, SE751 22 Uppsala, Sweden; 3 School of Health Sciences, Faculty of Medicine and Health, Örebro University, S701 82 Örebro, Sweden; 4 Unit of Public Health and Statistics, Region Östergötland, S581 85 Linköping, Sweden

**Keywords:** Involvement, Patient participation, Patient preferences, Person-centred care, Shared decision-making, Questionnaire, Survey

## Abstract

**Background:**

Although patient participation is strongly associated with high quality of healthcare, valid means to measure and report a comprehensive notion of patient participation are scarce. The Patient Preferences for Patient Participation (4Ps) is a new healthcare practice and research tool, comprising patients’ preferences as well as experiences. The 4Ps employs 12 items for the patient to conceptualise patient participation. The aim of this paper is to describe how the two perspectives of patient participation, namely preferences and experiences, can be combined to visualise and report preference-based patient participation.

**Methods:**

With four response alternatives in each section, the 4Ps offers sixteen possible combinations of degree of match per item. Theoretical and clinical principles fostered a tentative order of six ranks and three levels of preference-based patient participation. To test the standard, statistical analyses for ordinal data were performed, using data from a randomised controlled trial evaluating an intervention aiming to improve patient participation. Further, structures for visualising the preference-based patient participation of individuals and groups were suggested.

**Results:**

Data from the 4Ps demonstrated the individuals’ preference-based patient participation, indicating either a match or a mismatch for each item. Mismatches represented either the experience of participation surpassing the patient’s preferences, or the patient’s preferences for patient participation not being established. At group level, the suggested approach for visualising and reporting the 4Ps demonstrated that the intervention group had a significantly higher proportion of sufficient preference-based patient participation for certain items than the control group. These results had not been identified earlier, when using the preferences and experiences of patient participation as separate measures.

**Conclusions:**

Ways to easily acquaint stakeholders with patients’ preferences for patient participation are needed, in order for healthcare staff to better use resources to match the basic requirements of individuals and groups. While the 4Ps can guide professionals to patient participation as framed in legislations, concept analyses and by patients, a visualisation of the results is needed to capture preference-based patient participation. The proposed route to representing degree of match in preferences and experiences may also be relevant to other dimensions of quality of healthcare.

## Background

Modern healthcare allies with patient participation [1], although there is no single, common understanding of the concept [2–4]. Healthcare professionals often recount patient participation as related mainly to being engaged in decision-making [5], while patients depict it in a broader sense, including sharing experiences and information, shared decision-making, and self-management [6]. Furthermore, patients’ priorities for participation have been found to vary: the way in which and to what extent one prefers to partake can alter with, for example, type of healthcare contact and the reason for it [6, 7].

Just as patient participation is agreed to be essential for just and safe healthcare [8], valid means to capture variations and thus measure and report a comprehensive notion of patient participation are scarce [9]. With different attributes for patient participation at hand, instruments should incorporate all dimensions available, offering opportunities for patients to illustrate their experience in juxtaposition to their preferences. This is vital for assessing the quality of care and improvement opportunities [10].

For many quality aspects of healthcare, measures are fairly straightforward, targeting a ‘good’ experience (like ‘good access’ or ‘good services’). Yet, for a thick concept like patient participation, the complexity is more pronounced, and ‘good’ in terms of patient participation can fluctuate. For example, being engaged in healthcare decision-making can represent ‘good’ participation to a patient at a certain point – while for another individual, or the same person in a different situation, being expected to engage in a healthcare decision can represent ‘bad’ participation [11].

Thus, to explicitly measure and report healthcare quality in terms of patient participation, patient preferences for participation need to be considered alongside experiences. The 4Ps tool – standing for *Patient Preferences for Patient Participation* – is such a tool, developed by the first author based on a series of qualitative and quantitative studies on patients’ preferences for and experiences of patient participation [6, 11–15]. In former validation studies, the 4Ps has been found easy to comprehend and of conceptual clarity, whilst promising for measuring and evaluating patient participation in clinical practice and research [16, 17].

The 4Ps applies 12 attributes that have been found to illustrate and exhaust patient participation as a concept [18]. These attributes are framed as items, echoed in the tool’s two sections: a section for depicting one’s preferences, i.e. the importance of each item for satisfactory participation, and one for experiences, that is, the extent to which one has experienced the attributes of patient participation. The two sections of the 4Ps employ four response options each:

- For patient preferences, the response options for each item are that it is: 1) unimportant; 2) somewhat important; 3) very important, or; 4) crucial (for patient participation).
- For patient experiences, response options are that the attribute (of patient participation) presented in the item has been experienced: 1) not at all; 2) to some extent; 3) to a large extent, or; 4) entirely [18].

Used within a clinical context, the 4Ps is proposed to stimulate mutual understanding between staff and patient: the patient completes the preferences section at the onset of a healthcare interaction and the experiences section at a later, agreed point. This allows for the healthcare professional(s) and patient to jointly assess the individual’s preferences and experiences, respectively, and, most importantly, the match (or mismatch) between the patient’s experiences in relation to his or her preferences – hence evaluating ‘preference-based patient participation’. From a clinical perspective, this serves to appraise the conditions provided for patient participation; such comparisons can also be gauged at a group level, providing an illustration of, for example, how a team or unit performs in terms of preference-based patient participation. For research purposes, the 4Ps’ sections are to be used concurrently; this permits discrete appraisals of patients’ preferences and experiences, respectively, along with evaluations of the level of match between their experiences in relation to their preferences. Thus, the 4Ps can illustrate differences between, for example, groups of patients or settings, or the effects of a clinical intervention on preference-based patient participation.

However, this require further means for how to analyse, visualise, and thus represent measures of participation, recognising ordinal data vis-à-vis experiences and preferences, including the match between the two. To date, there are few (if any) examples of such enterprises. In a previous study, the 4Ps was employed to evaluate the effects on patient participation of a self-management support programme in primary care for patients with chronic heart failure (CHF) or chronic obstructive pulmonary disease (COPD) [19], in addition to the primary outcome self-efficacy. The data on patient participation was analysed by summarising each participant’s preferences and experiences, respectively, and the results of the two measures were presented separately at group level. While a Rasch analysis served as a means for summarising person measures from the data for each section [20], this procedure did not permit the detection of the level of match between preferences and experiences. Further, the above methods failed to depict any of the enhanced conditions for patient participation transpiring in a qualitative follow-up of the same study [21].

To capture patients’ experiences of participation in accord with patient preferences, tools such as the 4Ps are needed. Yet, in order to fully represent patients’ experiences and preferences, that is juxtaposing exclusive response options which yet communicate one and the same concept, guidelines on how to create a combined measure are commended. The aim of this paper is to describe how the two perspectives of patient participation, namely preferences and experiences, can be combined to visualise and report preference-based patient participation.

## Methods

### Setting and sample

This investigation employs the dataset on patient participation of the above study, where the 4Ps tool was applied for evaluative purposes [20]:

The data was collected between 2013 and 2015 in nine primary care centres across three regions in Sweden [19]; 79 patients were included in the intervention group and 83 in the control group. The intervention group had been block randomised for a self-management intervention programme: six 90-min group sessions every second week, led by a trained district nurse and a physiotherapist, addressing individual action plans and goals, and support for behavioural changes. Standard primary care was provided for both intervention and control group patients, the latter receiving no additional intervention during the study.

Patient preferences for and experiences of participation were measured using the 4Ps before and after the three-month intervention period [20]. The 4Ps was administered and collated along with assorted outcome measures, by an assigned nurse or physiotherapist separate from the intervention and blinded to the patients’ allocation to intervention or control group. The analysis is based on the preferences before the intervention and the experiences after.

### Development of a match-rank for preference-based patient participation

Given the 4Ps’ four response alternatives in two sections, there are 16 possible combinations in terms of the degree of match between a patient’s preference and experience for each item. To demarcate the different levels of preference-based patient participation, a ranking for the degree of match was established through a deliberate process guided by two empirical principles:

1. The closer the match, the better.
2. It is better to get more than you preferred, than less.

The principles descend from both previous studies on patient participation [6, 7, 11–17, 21] and healthcare norms, literally transpiring through F. Nightingale’s momentum to provide healthcare services of high quality yet in accordance with the desires of the patient [22], to today’s mutual engagement of the patient and the healthcare professionals on the patient’s team by means of for example person-centred care [23].

The principles signified that the 16 combinations of degree of match (between patient preferences and experiences for patient participation) could be categorised into six different ranks, ranging from zero to five. To illustrate, according to the principles the four combinations that represent a complete match all represent the top rank (rank five). Consequently, for example, the rank of an item with preference *Crucial* and experience *Entirely* is equivalent to the rank of an item with preference *Unimportant* and experience *Not at all*. These both represent rank five, that is a top match while an item with preference *Crucial* and experience *Not at all* represents the lowest possible rank, zero.

Furthermore, in order to provide further guidance with reference to patient participation in relation to quality of care, the ranks of preference-based patient participation were classified into three levels:

- ***Insufficient*** provision of preference-based patient participation, calling for significant progress (ranks 0–1),
- ***Fair*** provision, indicating room and need for improvement (ranks 2–3), and
- ***Sufficient*** provision, signifying complete or near match of patient preferences and experiences (ranks 4–5).

**Fig. 1:**
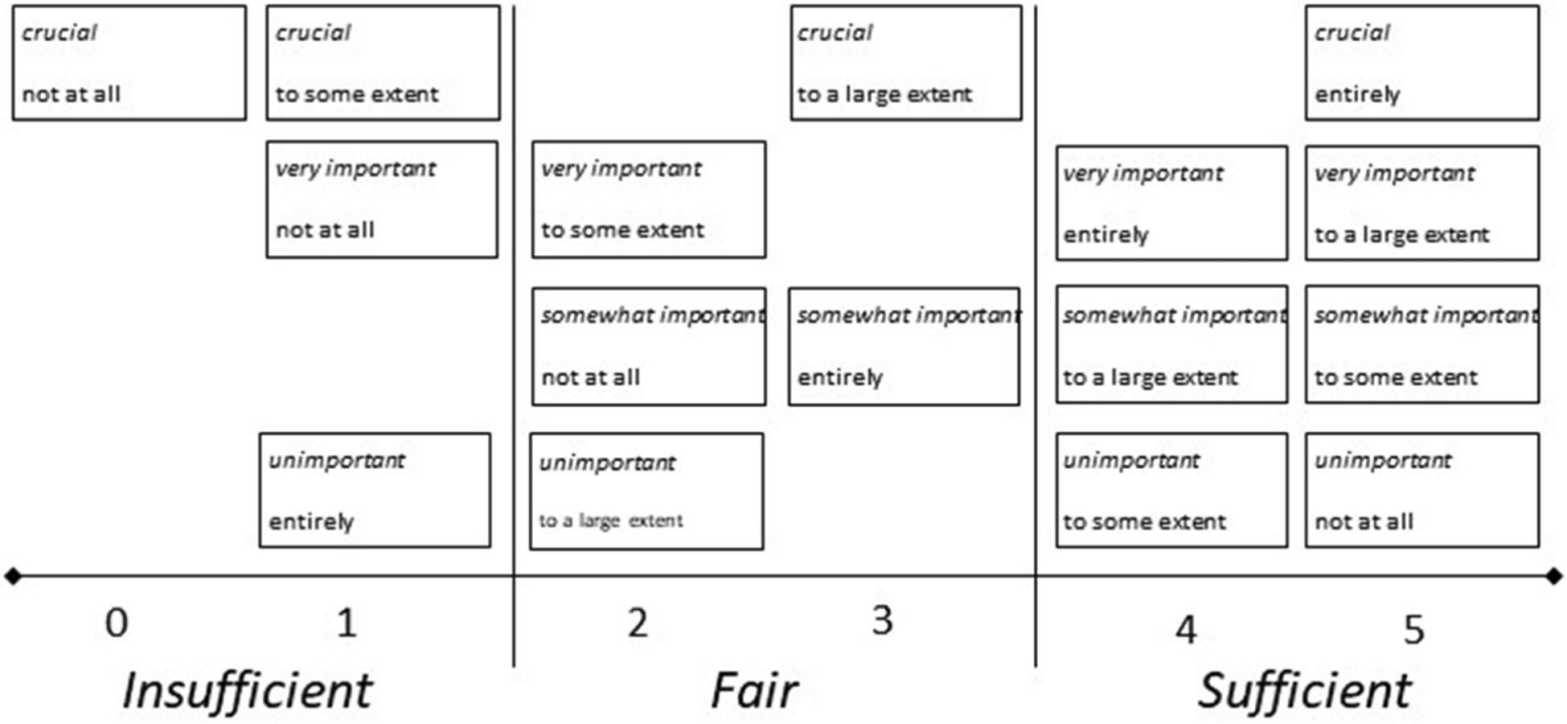
Overview of the 16 combinations for match (or mismatch) between patient preferences and experiences of patient participation (with preferences in italics and experiences in roman text) along with the ranks (0–5) and the three levels of preference-based patient participation

These levels were derived by an empirical approach, suggested to guide clinicians as well as scholars in further deliberating what is ‘good’ (or less appropriate) quality of care in terms of preference-based patient participation. For an overview of the 16 combinations of degree of match, the six ranks and the three levels of preference-based patient participation, see Fig. 1.

### Visualising and reporting of data

The data from the above study [20] were employed to visualise the outcomes in accordance with the match-rank between the preferences before and the experiences after the intervention. To start, individual profiles were formed, electing two individuals from the data set to illustrate the procedure and results. For the reporting of individual profiles, symbols were proposed to visualise each participant’s preference-based patient participation in relation to insufficient, fair or sufficient provision of preference-based patient participation. While this route is primarily proposed for clinical purposes, it may provide additional value to exemplify research results.

Furthermore, an analysis was performed to report whether, and to what extent, the outcomes indicate a difference between intervention and control groups in terms of patient participation in the trial, by means of the match-ranking. This was completed using patient group profiles. In the patient group profiling, the percentage of group members who fell into each of the three levels of preference-based patient participation is reported, using a figure at item level. This includes comparisons of proportions at group level, with chi-square tests between intervention and control groups. This is essentially proposed for research purposes but can serve a more comprehensive evaluation of quality of care.

As noted, although these profiles predominantly apply to clinical and research employment of the 4Ps tool, respectively, they can be used to serve either purpose. Rather, they are supposedly imperative for visualising and reporting the outcomes of the 4Ps.

## Results

### Preference-based patient participation – individual profiles

Preference-based patient participation for individuals is represented as a chart, including the actual ratings of preferences (P) and experiences (E), respectively. These are placed in four corresponding columns in the table, where symbols indicate the classified level of the rank. This suffices to provide an image demonstrating whether a mismatch signified that the experiences were rated as lower or higher than the individual’s preferences. Examples of the reporting of two individuals are shown in Table 1, illustrating that patient 18’s data showed a pattern where only the conditions for reciprocal communication were deemed sufficient, with a complete match between preference and experience. All other items showed less participation than preferred; in four items there was an almost complete divergence, with the preference for patient participation being “crucial” and the experience only “to some extent”. Patient 66 data, on the other hand, illustrated sufficient preference-based patient participation for all but one aspect. However, a complete match occurred only for one item (“managing treatment”). For “learning of plans”, the one item assigned as crucial for patient participation, the experienced participation was less than the individual’s preference.

**Table 1:** Patient profile for preference-based patient participation for patient 18 and patient 66. Response options for patient preferences (**P**): 1 = unimportant, 2 = somewhat important, 3 = very important, and 4 = crucial (for patient participation), and for patient experiences of patient participation (**E**): 1 = not at all, 2 = to some extent, 3 = to a large extent, and 4 = entirely. Levels of match between preferences and experiences: insufficient = **X**, fair =**!**, and sufficient = **√**

Items	Patient 18					Patient 66				
	Response option				Level	Response option				Level
	1	2	3	4		1	2	3	4	
1. Being listened to			**E**	**P**	**!**		**P**	**E**		**√**
2. Experiences being recognised		**E**		**P**	**X**		**P**	**E**		**√**
3. Reciprocal communication			**PE**		**√**		**P**	**E**		**√**
4. Sharing one’s symptoms			**E**	**P**	**!**		**P**	**E**		**√**
5. Explanations as to symptoms		**E**		**P**	**X**		**P**	**E**		**√**
6. Being told what is done		**E**	**P**		**!**		**P**	**E**		**√**
7. Learning of plans			**E**	**P**	**!**			**E**	**P**	**!**
8. Taking part in planning		**E**		**P**	**X**		**P**	**E**		**√**
9. Phrasing personal goals		**E**	**P**		**!**		**P**	**E**		**√**
10. Learning to manage symptoms		**E**		**P**	**X**		**P**	**E**		**√**
11. Managing treatment			**E**	**P**	**!**			**PE**		**√**
12. Managing self-care			**E**	**P**	**!**		**P**	**E**		**√**

### Preference-based patient participation – group profiles

**Fig. 2:**
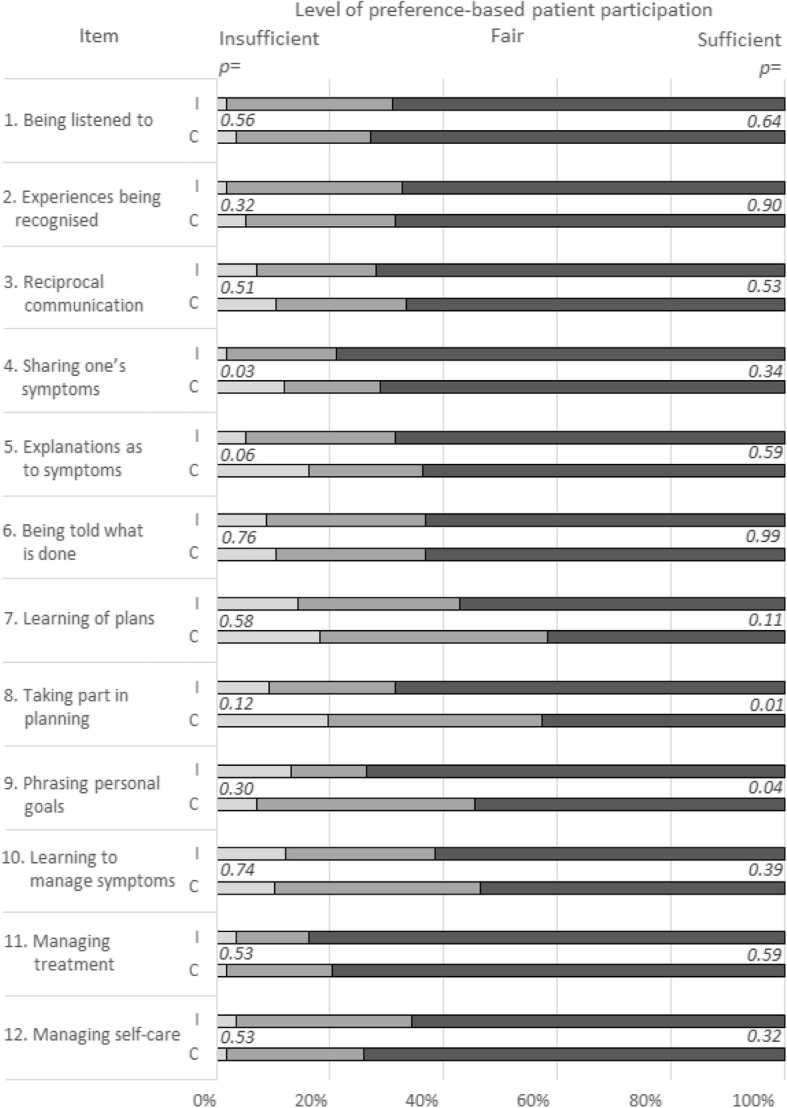
Patient group profiles at the level of preference-based patient participation, including intervention (I) and control (C) groups in the RCT study on patient participation. The *p*-values (significance level *p* = 0.05) are reported in relation to insufficient and sufficient preference-based patient participation, respectively

Analysing the data from the above study from a preference-based patient participation perspective at group level, the intervention group was found to have more preference-based patient participation in certain items (see Fig. 2). For example, the match-rank indicated that the experiences of the intervention group to a larger extent matched their preferences in terms of “taking part in planning” (69 versus 43% of the participants having a sufficient match, *p* = 0.01) and “phrasing personal goals” (74 versus 55% with a sufficient match, *p* = 0.04), when compared to the control group. In addition, for three items, in the control group there were at least 10 percentage points more of insufficient match between preferences for and experiences of patient participation. For example, in item 4 “Sharing one’s symptoms”, 12% of patients in the control group had an insufficient match compared to 2% of patients in the intervention group (*p* = 0.033).

## Discussion

When investigating a concept like patient participation, which is considered significant and often laden with positive connotations even if not thoroughly seized, insight into patients’ preferences is crucial; in order to provide for experiences that are significant to patients, the experiences need to match the notion that applies in a particular healthcare setting and/or at a particular time point. We propose this represents preference-based patient participation. The method of examining, reporting and visualising preference-based patient participation proposed in this paper offers a novel approach; while there are particular quality of care assessments including both to what extent patients have perceived certain aspects of care and how important they consider these to be [24, 25], the 4Ps offers opportunities both to prospect patients’ preferences for patient participation and to evaluate to what level patient experiences match these preferences – indicating to what extent there are conditions for patient participation that align with standards [18]. The match between preferences and experiences represents whether participation has been good or not – and reveals any need for improvements to procure sufficient quality of care. While the structure and content of the 4Ps can suffice conditions for patient participation, based on an improved understanding of the patient’s voice and choice, as both individuals and groups, further studies are suggested to identify the ideal actions, actors, and points of care and time for employing the 4Ps [18].

For now, as the 4Ps tool does not provide a common route whereby a higher degree of all types of participation is consequentially better, a match-rank between preferences and experiences is important in order to visualise what constitutes ‘good’ and ‘bad’ patient participation, respectively [26, 27]. By using a match-rank, the fit of the participation experienced by the patient relative to the preference profile is measured, making it possible to illustrate quality of care from the perspective of both individual and group-level data [28]. A summarised, Rasch-based person measure for preferences and experiences respectively was employed in earlier reporting of the 4Ps as an outcome measure [20]. This summarised score, although clear and easy to use in statistical analyses, comes with a disadvantage: single scores are associated with an assumption that more is better, although it did not take the individuals’ preferences into account when evaluating their experiences. Rather, the alternative route applied here convey harmony with the qualitative follow-up of the same study, indicating that the intervention group experienced enhanced opportunities for taking responsibility for one’s own goals and assimilating the knowledge shared [21]. Thus, the measures applied in this secondary analysis can serve both scholars and clinicians to comprehend the 4Ps outcomes in terms of preference-based participation, both at an individual patient level and at a group level. Presumably, reporting and visualising the 4Ps results will also aid the identification of needs for improvement with regard to patient participation.

Opportunities to easily acquaint healthcare professionals with patients’ primary preferences are scarce, although this would reveal how healthcare staff can better use resources to match the basic requirements of individuals and groups [29]. A match between an individual’s preferences for participation and experienced participation supposedly indicates perceptions of a high quality of care; consequently, supporting patients in attaining their preferred level of participation might be of greater importance to them than increasing their desire to enact participation by means of activities and decisions [30]. A tool like the 4Ps can also aid patients who are less prone to voice their preferences [31], providing them with an opportunity to obtain the kind and level of participation they prefer, aiding them in conceptualising and sharing preferences, guided by what is semantically and judicially framed as participation.

The visualisation of patterns in what is expected in terms of opportunities for patient participation and what works to support matching experiences can inform quality-improvement initiatives. With opportunities to repeat both sections of the 4Ps, this may well show that, over time, both preferences and experiences alter, potentially with an improved match as dialogues and awareness increase [32]. Furthermore, with the 4Ps and the visualisations and reporting standards suggested, the results of particular clinical interventions can be compared between subgroups, units, departments, hospitals or regions. Findings can also be evaluated over time. However, with significant differences noted at the item level, rather than in sum scores, the data from the 4Ps necessitates an item level [33]. This is essential to recognise, because differences between small groups need to be detected for example, when evaluating preference-based patient participation within departments or patient groups. In addition, further studies are needed to understand if certain preferences for patient participation are easier to meet than others, and whether this account for particular or all attributes.

In the study using summarised person measures for one section at a time, which used the same data set, no significant differences between intervention and control groups were identified [20]. When using preference-based ranks, a more differentiated result emerged, showing that there were some items where preference-based participation was better in the intervention group than the control group. This indicates the possibility of the intervention having an effect on some aspects of patient participation, that was concealed when using summarized measures and not relating experiences to preferences on an individual basis.

While patient participation is still habitually associated with engagement in decision-making regarding one’s own health and healthcare, the voices of patients signify a wider conceptualisation, incorporating attributes depicted in the 4Ps relating to shared decision-making, patient-centred care, person-centred care and self-management [18, 34]. Thus, the primary notion of participation as in ‘sharing of’ (usually experiences, knowledge and information) and ‘sharing in’ (actions related to healthcare and self-care) seems vital for good care [35]. Clinical employment of the 4Ps requires balancing individuals’ recognition of preferences with experiences at group and service levels, although prospects for a shared understanding of expectations and conditions are likely valuable for both patients and professionals. Still, introducing opportunities to share a combined measure of preferences for and experiences of patient participation in healthcare dialogues is novel, and like most new procedures, applications of the 4Ps in clinical settings are likely to entail some degree of effort [36]. Consequently, there is a need to investigate which strategies enable implementation of an innovation like the 4Ps, and whether and how this facilitates preference-based patient participation in different contexts [37–39].

## Conclusion

In this study, we have visualised preference-based patient participation, at both individual and group levels, with a focus on presenting results that are easily interpreted and meaningful to both patients and healthcare staff, as well as scientists. The route to presenting and visualising a match in preferences and experiences may also be relevant to other dimensions of quality in healthcare, suggesting means additional to for example the Rasch person measure in cases where the concept investigated requires a combination of preferences and experiences to indicate whether the outcome is good, or any level of less fitting.

## Data Availability

The datasets used and/or analysed during the current study are available from the corresponding author on reasonable request.
